# Cortical tau load is associated with white matter hyperintensities

**DOI:** 10.1186/s40478-015-0240-0

**Published:** 2015-09-30

**Authors:** Kirsty E. McAleese, Michael Firbank, Madhurima Dey, Sean J. Colloby, Lauren Walker, Mary Johnson, Joshua R. Beverley, John Paul Taylor, Alan J. Thomas, John T. O’Brien, Johannes Attems

**Affiliations:** Institute of Neuroscience, Newcastle University, Campus for Ageing and Vitality, Newcastle upon Tyne, NE4 5PL UK; Department of Psychiatry, Cambridge University, Cambridge, UK

**Keywords:** Hyperphosphorylated tau, White matter hyperintensities, White matter lesions, Small vessel disease, Alzheimer’s disease, *Post-mortem* MRI

## Abstract

**Introduction:**

Cerebral white matter lesions (WML), visualized as white matter hyperintensities (WMH) on T2-weighted MRI, encompass structural damage and loss of integrity of the cerebral white matter (WM) and are commonly assumed to be associated with small vessel disease (SVD). However, it has been suggested that WM damage may also be the result of degenerative axonal loss that is secondary to cortical Alzheimer’s disease (AD) pathologies i.e., hyperphosphorylated tau (HPτ) and amyloid-beta (Aβ). Here we investigate the influence of HPτ, Aβ and SVD on WMH severity.

**Results:**

36 human *post-mortem* right fixed cerebral hemispheres (mean age 84.4 ± 7.7 years; male: 16, female: 20) containing varying amounts of AD-pathology (AD: 23, controls: 13) underwent T2- weighted MRI with WMH assessed according to the age related white matter change scale (ARWMC). After dissection, using tissue samples from the frontal, temporal, parietal and occipital regions from the right hemisphere, we quantitatively assessed cortical HPτ and Aβ pathology burden by measuring the percentage area covered by AT8 immunoreactivity (HPτ-IR) and 4G8 immunoreactivity (Aβ-IR), and assessed the severity of WM SVD by calculating the sclerotic index (SI) of WM arteries/arterioles. HPτ-IR, Aβ-IR, and SI were compared with ARWMC scores. HPτ-IR, Aβ-IR and WM ARWMC scores were all significantly higher in AD cases compared to controls, while SI values were similar between groups. ARWMC scores correlated with HPτ-IR, Aβ-IR and SI in various regions, however, linear regression revealed that only HPτ-IR was a significant independent predictor of ARWMC scores.

**Conclusions:**

Here we have shown that increasing cortical HPτ burden independently predicted the severity of WMH indicating its potentially important role in the pathogenesis of WM damage. Moreover, our findings suggest that in AD patients the presence of WMH may indicate cortical AD-associated pathology rather than SVD. Further studies are warranted to elucidate the pathological processes that lead to WM damage and to clarify if WMH may serve as a general biomarker for cortical AD-associated pathology.

## Introduction

Cerebral white matter lesions (WML), as visualized histologically, encompass structural damage and loss of integrity of the cerebral white matter (WM) due to WM rarefaction (*i.e.,* demyelination and axonal loss), which is commonly accompanied by reactive astrocytosis and edema [21]. WML frequently occur in brains of both demented and non-demented elderly, and are visualized as white matter hyperintensities (WMH) on *pre-* and *post-mortem* T2-weighted magnetic resonance imaging (MRI) [22]. Age-associated deep WM changes are distinguishable as ‘punctate’, i.e., single lesions <10 mm, ‘early confluent’, i.e., single lesion <20 mm and/or multiple lesions 10–20 mm linked by ‘connecting bridges’, or ‘confluent’ changes, i.e., confluent area of damage >20 mm in diameter [52]. These lesions differ from that seen in multiple sclerosis (MS) as MS WML are typically ovoid in shape, range between 3-8 mm in diameter and are mainly located in the periventricular WM, posterior fossa and subcortical structures [7]. The Austrian Stroke prevention study indicated that the prevalence of WMH is between 62–96 % in individuals aged 45 to 87 years [51]. WMH are associated with a wide range of cognitive deficits, such as cognitive decline [54], and are a frequent co-pathology in Alzheimer’s disease (AD) [30], which is the most frequent cause of age-associated dementia [57]. The pathogenesis of WMH is typically associated with small vessel disease (SVD) of the WM [20], however; the pathogenic mechanisms underlying the development of WM damage are not well understood. Previous longitudinal, neuropathological and imaging studies suggest a multifactorial aetiology of WML [13, 16, 32, 33, 58, 62] including WM damage secondary to both SVD- related ischemia and cortical AD pathology, *i.e.,* depositions of intracellular hyperphosphorylated tau (HPτ) and extracellular amyloid-beta (Aβ). SVD alterations are assumed to lead to disturbed arterial autoregulation, promoting progressive stenosis that eventual leads to chronic hypoperfusion of the surrounding WM [26]. The exact pathological mechanisms of degenerative axonal loss is still unclear, but it is suggested axonal death occurs in conjunction with AD-pathology related grey matter atrophy, or via calpain-mediated degradation of cytoskeletal proteins, activated by AD pathology-related axonal transport dysfunction [9, 36]. However, currently neither imaging nor routine histological techniques allow for differentiation between ischemic (SVD-related) or neurodegenerative causes (AD pathology) of WM damage.

Previous studies investigating the relationship between WM damage with both cortical neurodegenerative pathology and WM SVD used semi-quantitative methods to evaluate the severity of the respective pathology [13, 24, 35]. However, such semi-quantitative methods provide only a crude estimation of the severity of pathology [2], while quantitative methods more accurately reflect the actual amount of pathology present. Therefore, we quantitatively measured cortical HPτ and Aβ burden, as well as the degree of vessel wall thickening of WM arteries/arterioles in human *post-mortem* brains, to determine the influence of both cortical AD pathology and SVD on WM integrity that was assessed using *post-mortem* T2-weighted MRI.

## Materials and methods

Our study cohort consisted of 36 human *post-mortem* brains (mean age 84.4 ± 7.7 years; male: 16, female: 20) with varying amounts of AD-pathology, which were clinico-pathologically classified as AD: 23, and controls: 13. Mini mental state examination (MMSE) [17] scores were available for 24 cases (AD: 18; controls: 6). Demographic and neuropathological characteristics of the study cohort are shown in Table 1. Brain tissue was obtained at autopsy and stored within the Newcastle Brain Tissue Resource (NBTR) in accordance with Newcastle University Ethics Board (The Joint Ethics Committee of Newcastle and North Tyneside Health Authority, reference: 08/H0906/136). After autopsy the right hemisphere, brainstem and cerebellum were immersion fixed in 4 % buffered aqueous formaldehyde solution for 6 weeks.

**Table 1:** Characteristics of study cohort

	AD	Control	Statistic (*df*, *p*-value)
*n*	23	13	
Age, mean (±SD)	84.26 years (5.67)	84.77 (8.49)	t_(34)_ = 1.194, *p* = 0.241
Gender M:F	10:13	8:7	*χ* ^2^_(1)_ = 0.444, *p* = 0.505
PMD, mean (±SD)	49.95, h (22.89)	47.15 (24.43)	t_(33)_ = 0.34, *p* = 0.735
Thal Aβ phase^20^	Phase 5, *n* = 23	Phase 0, *n* = 5	U_(34)_ = 0.000, *p* = 0.001
		Phase 1, *n* = 3	
		Phase 2, *n* = 3	
		Phase 3, *n* = 1	
		Phase 4, *n* = 1	
Braak NFT stage^21^	NFT stage 6, *n* = 23	NFT stage 0, *n* = 2	U_(34)_ = 0.000, *p* = 0.001
		NFT stage 1, *n* = 1	
		NFT stage 2, *n* = 3	
		NFT stage 3, *n* = 6	
		NFT stage 4, *n* = 1	
CERAD^22^	C, *n* = 23	Negative, *n* = 10	-
		A, *n* = 2	
		B, *n* = 1	
NIA-AA^23^	High, *n* = 23	No, *n* = 5	-
		Low, *n* = 7	
		Intermediate, *n* = 1	
MMSE^17^ (±SD)	4.33 (3.9)	28 (2.09)	t_(22)_ = 14.07, *p* = 0.001

*Abbreviations*: *AD* Alzheimer’s disease, *df* degrees of freedom, *t* Independent samples test, *X* ^*2*^ Chi -squared test, *F* female, *M* Male, *U* Mann–Whitney *U* test, *PMD post mortem* delay, *Aβ* amyloid-beta, *NFT* neurofibrillary tangle, *CERAD* Consortium to Establish a Registry for Alzheimer's Disease, *NIA-AA* National Institute on Ageing - Alzheimer’s Association criteria for AD neuropathologic change, *MMSE* mini mental state examination

### *Post-mortem* magnetic resonance imaging

We have previously demonstrated that *post-mortem* MRI of fixed hemispheres reliably reflect WM damage as accurately as an extensive histological assessment at 7 mm intervals [38]. Briefly, fixed right hemispheres were removed from formalin solution and were investigated using a 4.7 T MRI scanner (Bruker Medical, Ettlingen, Germany): Bruker Biospec 47/60 VAS, (vertical, actively shielded, the inner-bore width of 60 cm) fitted with a BGA-38-S gradient system (actively shielded, the inner-bore width of 38 cm) and a birdcage radio-frequency coil with a working cross-section of 170 × 240 mm. AT2-weighted pulse sequence was used: two spin echo images of effective echo time (TE) = 32/96 ms, repetition time (TR) = 8200 ms, with slice thickness of 2 mm and planar resolution of 1.0 × 0.78 mm. Regional WMH were subjectively rated, blinded to clinical diagnosis, by two experienced assessors (M.F & J.T.O) according to the age-related white matter change scale (ARWMC) [60]; score 0, absence of WMH; score 1, ‘punctate’ WMH (<10 mm); score 2, ‘early confluent’ WMH (<20 mm); score 3, ‘confluent’ WMH (>20 mm). ARWMC scores were generated for the frontal and temporal WM and a combined score for the parietal and occipital WM i.e., parieto-occipital. All ARWMC scores were combined to calculate mean total ARWMC score that reflected WMH severity of the entire hemisphere.

### Routine neuropathological assessment

Irrespective of clinical diagnoses, all brains underwent neuropathological assessment to standardized neuropathological scoring/grading systems, including Thal phases of Aβ deposition [55], Braak staging of neurofibrillary pathology [6], Consortium to Establish a Registry for Alzheimer’s Disease (CERAD) scores [40] and the National Institute on Aging-Alzheimer’s Association (NIA-AA) criteria [41] (Table 1).

### Tissue preparation

Six μm paraffin-embedded sections were cut from six cerebral regions; pre-frontal cortex (Brodmann area (BA) 10, 9), mid-frontal cortex (BA 8, 9), entorhinal cortex (BA 36, 28), temporal cortex (BA 36), parietal cortex (BA 40/22), and occipital cortex (BA 17). Tissue sections were mounted onto 4 % 3-aminopropyltriethoxysilane (APES)-coated glass slides and histologically stained with haematoxylin and eosin (H&E). Immunohistochemistry was performed for HPτ (antibody AT8; dilution 1:4000; Innogenetics, Ghent, Belgium) and Aβ peptide (clone 4G8; dilution 1:15,000; Signet Labs, Dedham, MA, USA). Prior to immunostaining, antigen retrieval was performed by microwaving slides in 0.01 mL citrate buffer for 10 min (AT8) or immersed for 1 hour in concentrated formic acid (4G8). Immunopositivity was detected using the Menarini X-Cell-Plus HRP Detection Kit (Menarini Diagnostics, Winnersh-Wokingham, UK) with 3,3 diaminobezidine (DAB) as a chromagen and haematoxylin as a counter stain. Sections were subsequently dehydrated through a series of alcohols, cleared and mounted using DPX (CellPath, Powys, UK).

### Quantification of protein aggregates - image analysis

**Fig. 1:**
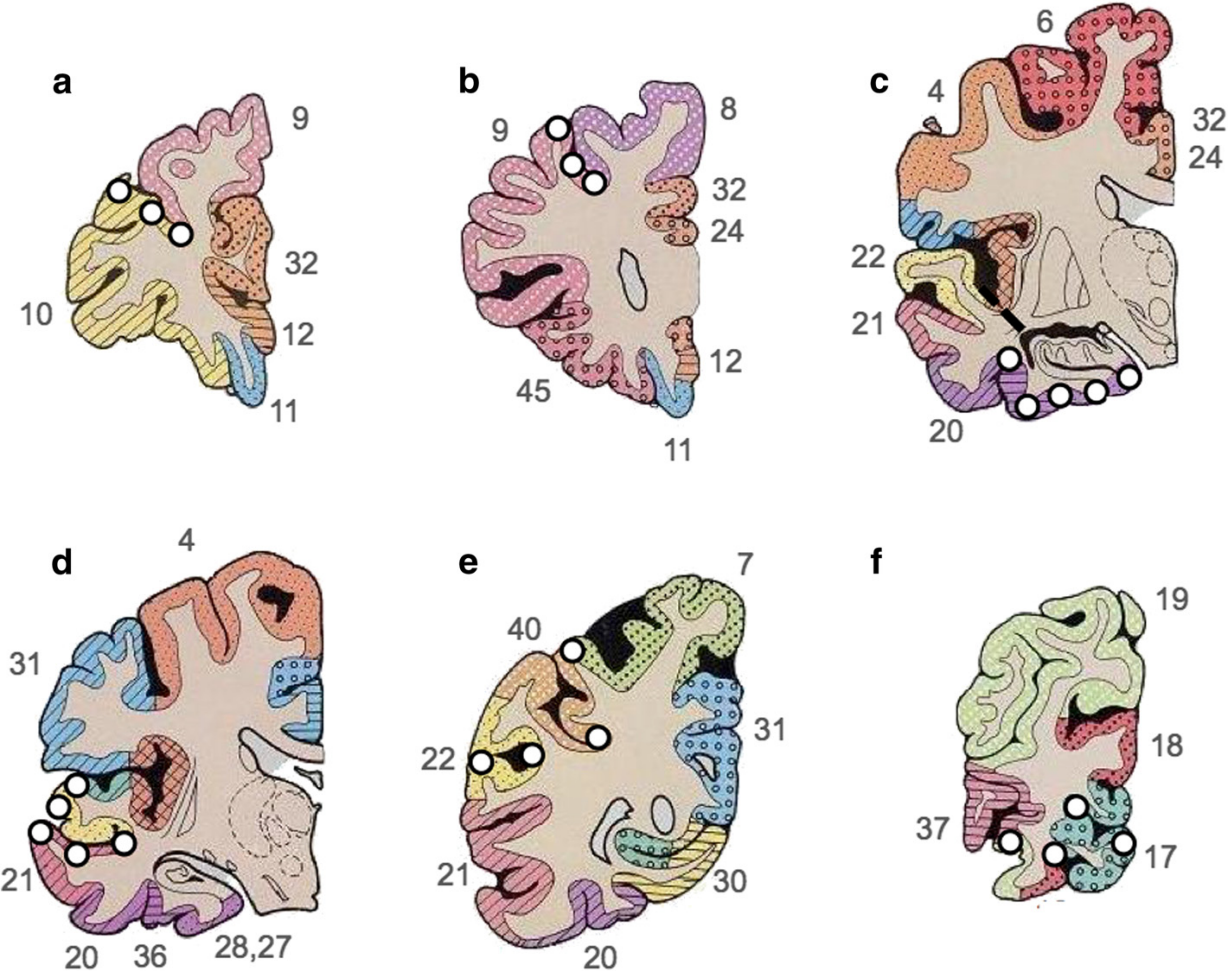
Cartoon illustrating the 24 locations used for image analysis encompassing sulci, midsection, and gyri from prefrontal (**a**), mid frontal (**b**), entorhinal (**c**), temporal (**d**), parietal (**e**) and occipital (**f**) cortices. White dots indicate the sampling location; numbers refer to Brodman areas, which are marked with coloured patterns (modified from [46])

Image analysis was performed blinded to neuropathological diagnosis. On tissue sections immunostained for AT8 and 4G8, 24 cortical sample areas were identified for analysis. Sample location included sulci, midsection and gyri in order to yield an accurate mean measure of cortical pathological burden as protein aggregates have been shown to be highest in the sulcus and lowest in the gyral tip [18, 39] (Fig. 1). For image analysis, a Nikon 90i microscope coupled to a PC with NIS-Elements AR3.2 software (Nikon, Surrey, UK) was used. At each sample location 3x3 single images were captured at 200x magnification and combined to create one large image representing an area of 1.7 mm^2^. If necessary, large images were subjected to manual setting of regions of interest to exclude WM and meningeal structures. Pixels in the binary layer were used to measure immunoreactivity (IR). Red Green Blue (RGB) thresholds for binary layer pixels were standardized separately for AT8 and 4G8 and set at a level that was reached by immunopositive pathological structures only (i.e., neurofibrillary tangles (NFT), neuropil threads, and Aβ plaques). RGB intensity values are measured on a scale between 0 and 255 (see NIS elements version 3.0, user guide, 2008, Nikon, Surrey, UK) and were set as follows; AT8: R25-170, G27-156, B11-126; 4G8: R50-180, G20-168, B8-139. In addition to RGB thresholds, we also set a size restriction threshold for the assessment of 4G8, which excluded the measurement of immunoreactive signals with an area below 100 μm^2^; this was necessary to ensure that physiological APP that is stained with 4G8 antibody was not included, and did not contribute to false positive values. The area covered by IR was stated as the percentage of the total measured area and the respective values are expressed as HPτ-IR (AT8) and Aβ-IR (4G8). Mean regional values for frontal, temporal (including the entorhinal cortex), parietal and occipital cortical HPτ-IR and Aβ-IR were calculated. Regional HPτ-IR and Aβ-IR values were combined to calculate mean total HPτ- and Aβ-IR values, respectively, which reflected cortical pathological burden of the entire hemisphere.

### Sclerotic index

The sclerotic index (SI) is a quantitative measure of arterial and arteriolar vessel wall thickness and has been shown to be an accurate indicator for the severity of SVD [31, 61]. The standard formulae SI = 1 - (internal diameter/external diameter) was used; the SI of normal arteries and arterioles ranges from 0.2 to 0.3, while an SI of 0.3 to 0.5 indicates mild to moderate SVD and SI values >0.5 are seen in severe SVD [31]. On H&E stained tissue sections, adjacent to the ones used to determine HPτ-IR (AT8) and Aβ-IR (4G8; Fig. 1), the SI of WM vessels was examined, blinded to neuropathological diagnosis, using a Nikon 90i microscope at 200x magnification. Eight randomly selected cerebral WM arteries and/or arterioles >50 μm diameters were identified per section and a single image captured using a DsFi1 camera. SI was calculated using the software program VasCalc as previously described [61]. SI values from the eight individual vessels from temporal, parietal and occipital WM were used to calculate mean values for each WM region. Eight individual SI values from both the pre- and mid-frontal WM were combined and used to calculate a mean value for the frontal WM. All regional SI scores were taken to calculate a mean total SI value to reflect the severity of SVD in the entire hemispherical WM.

### Statistical analysis

The Statistical Package for Social Sciences software (SPSS ver. 21) was used for statistical evaluation. Variables were tested for normality using the Shapiro-Wilk test and visual inspection of variable histograms. Subsequently, group effects were assessed using either non-parametric (Mann–Whitney U) or parametric procedures (independent samples test). Where appropriate, partial Spearman’s (ρ') and Pearson’s (r') correlation coefficients (one tailed) were used to assess associations between ARWMC scores and HPτ-IR, Aβ-IR and SI (controlling for the effects of age at death). Exploratory forward stepwise linear regression analyses were also conducted to investigate predictors of ARWMC scores.

## Results

### Differences between total cortical HPτ/Aβ burden, total WM SVD severity and severity of WMH between AD and controls

**Fig. 2:**
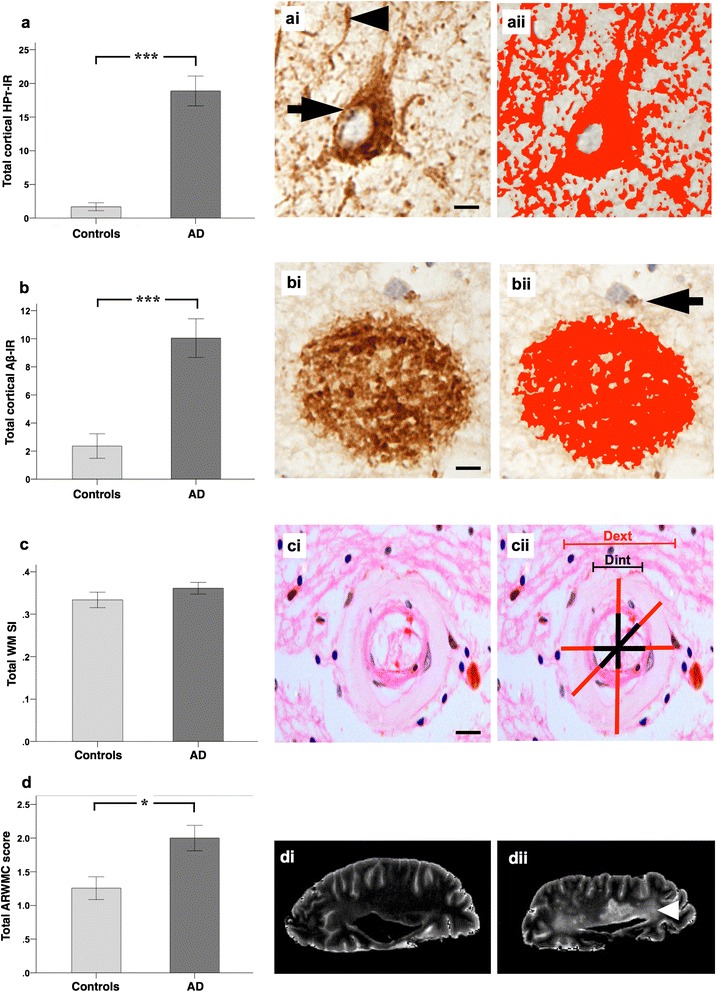
Total cortical percentage area covered by HPτ-IR (AT8 antibody for hyperphosphorylated tau burden) and 4G8 immunoreactivity (Aβ-IR; for amyloid-β burden), total white matter sclerotic index (WM SI; measure of small vessel disease severity), and total age related white matter change scale (ARWMC; measure for white matter hyperintensities) scores in Alzheimer’s disease (AD) i.e., cognitively impaired, and controls. **a**, total HPτ-IR is significantly higher in AD as compared to controls. (*ai*), hyperphosphorylated tau in the form of neurofibrillary tangles and neuropil threads is immunopositive for AT8 antibody (neurofibrillary tangle - arrow, neuropil thread - arrow head) and (*aii*) the area covered by AT8 immunopositivity (shaded in red) was measured to calculate HPτ-IR. **b**, total Aβ-IR was significantly higher in AD as compared to controls. (*bi*), Aβ plaques/depositions are immunopositive for 4G8 antibody and (*bii*) the area covered by 4G8 immunopositivity (shaded in red; arrow indicates physiological APP that is not included in the analysis) was measured to calculate Aβ-IR. **c**, No differences were seen in the severity of small vessel disease as measured by sclerotic index (SI) between controls and AD. (*ci*), small white matter artery, histologically stained with H&E. (*cii*) example for calculating the SI: using VasCalc software [61] the internal (Dint) and external (Dext) diameters were measured three times to yield a SI value (for details see main text). **d**, Total age related white matter change (ARWMC) scores [60] were significantly higher in AD compared to controls. Examples of *post mortem* T2 MRI: (*di*), no WMH (ARWMC score 0) and (*dii*) confluent white matter hyper intensity in the parieto-occipital region (arrow head; ARWMC score 3). MR images were captured in sagittal plane. *,*p* < 0.05; ***,*p* < 0.001; scale bar, 20 μm, valid for *ai* and *bi*, 50 μm for *ci*

As expected, total cortical HPτ-IR was significantly higher in cases clinico-pathologically classified as AD compared to controls (*p* = 0.001, Fig. 2a), as was total cortical Aβ-IR (*p* = 0.001, Fig. 2b). Total WM SI values did not differ significantly between AD and controls (*p* = 0.241, Fig. 2c), while total ARWMC scores were also significantly higher in AD relative to controls (*p* = 0.031, Fig. 2d).

### Correlations between cortical HPτ/Aβ burden and WM SVD severity with severity of WMH

**Fig. 3:**
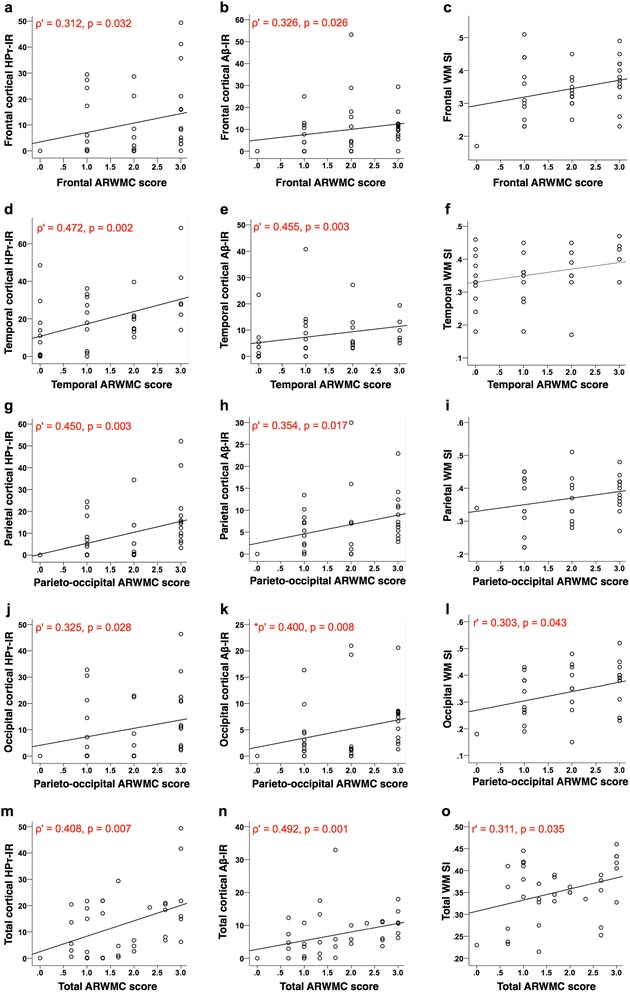
Partial Spearman’s (ρ') (HPτ-IR and Aβ-IR) and Pearson’s (r') (WM SI) correlation coefficients controlling for the effect of age at death. Scatter graphs show correlations between cortical percentage area covered by HPτ-IR (AT8 antibody for hyperphosphorylated tau burden), Aβ-IR (4G8; for amyloid-β depositions/plaques) and total white matter sclerotic index (WM SI; measure of small vessel disease severity) with age related white matter change score (ARWMC; measure for white matter hyperintensities) in the frontal (**a**-**c**), temporal (**d**-**f**), parietal (**g**-**i**) and occipital (**j**-**l**) regions, as well as total values representing the entire hemisphere (**m**-**o**). Analysis revealed that in all regions, and the entire hemisphere, significant correlations were observed between ARWMC scores and cortical HPτ-IR and Aβ-IR values, respectively. A significant relationship between ARWMC scores and WM SI were observed only in the occipital lobe. Of note, a correlation was observed between total SI and total ARWMC scores but this is likely due to the correlation in the occipital region. Only significant p values (and associated correlation coefficients (ρ’, r’)) are shown; for all correlation please see main text

To investigate the associations between cortical HPτ/Aβ burden and WM SVD severity with WMH scores, we performed partial Spearman’s (ρ') and Pearson’s (r') correlation analyses, controlling for age at death, across various topographic regions and the entire brain (Fig. 3). Analysis revealed that cortical HPτ-IR values correlated with ARWMC scores in all regions; frontal, ρ' = 0.312, *p* = 0.032; temporal, ρ' = 0.472, *p* = 0.002; parietal, ρ' = 0.450, *p* = 0.003 and occipital ρ' = 0.325, *p* = 0.028, as well as the entire hemisphere (ρ' = 0.408, *p* = 0.007). Correlations were also observed between ARWMC scores and cortical Aβ-IR values in all regions and for the entire hemisphere; frontal, ρ' = 0.326, *p* = 0.026; temporal, ρ' = 0.455, *p* = 0.003; parietal, ρ' = 0.354, *p* = 0.017; occipital, ρ' = 0.400, *p* = 0.008; total, ρ' = 0.492, *p* = 0.001. However, correlations between ARWMC scores and WM SI were observed only in the occipital lobe (r' = 0.303, *p* = 0.043) and not the frontal (r' = 0.273, *p* = 0.056), temporal (r' = 0.284, *p* = 0.061) or parietal (r' = 0.210, *p* = 0.113) regions. A correlation was revealed between total values of ARWMC scores and WM SI (r' = 0.311, *p* = 0.035), however this is likely due to the correlation in the occipital region.

### Neuropathological predictors of WMH

To investigate whether the burden of cortical neurodegenerative pathology (i.e., HPτ and Aβ) and severity of SVD in the WM independently predicted WMH score, stepwise linear regression analyses were performed for each region with ARWMC scores as the dependent variables and cortical HPτ-IR, cortical Aβ-IR, WM SI scores and age at death as independent variables.

In the temporal region, only HPτ-IR was a significant predictor (model R^2^ = 0.270, F_(2)_ = 5.359, *p* < 0.001; β = 0.532, *p* = 0.003) while the remaining independent variables were not significant predictors (cortical Aβ-IR: β = 0.081, *p* = 0.087; WM SI: β = 0.306, *p* = 0.358; age at death: β = 0.194, *p* = 0.245). With respect to the frontal and parietal regions, both cortical HPτ-IR (frontal: model R^2^ = 0.208, F_(2)_ = 4.32, *p* < 0.001; β = 0.340, *p* = 0.022; parietal: model R^2^ = 0.280, F_(2)_ = 6.410, *p* < 0.001; β = 0.417, *p* = 0.009) and age at death (frontal: β = 0.398, *p* = 0.018; parietal: β = 0.384, *p* = 0.015) were significant predictors but not cortical Aβ-IR (frontal: β = 0.059, *p* = 0.725; parietal: β = 0.068, *p* = 0.068) nor WM SI (frontal: β = 0.204, *p* = 0.248; parietal: β = 0.129, *p* = 0.149). In the occipital regions, only age at death was a significant predictor (model R^2^ = 0.135, F_(1)_ = 4.819, *p* < 0.001; β = 0.367, *p* = 0.036) but not HPτ-IR (β = 0.297, *p* = 0.081), Aβ-IR (β = 0.266, *p* = 0.114) or WM SI (β = 0.147, *p* = 0.252). With respect to the entire hemisphere, total cortical HPτ-IR (model R^2^ = 0.331, F_(2)_ = 8.162, *p* < 0.001; β = 0.514, *p* = 0.001) and age at death (β = 0.386, *p* = 0.012) were significant predictors but not total cortical Aβ-IR (β = 0.074, *p* = 0.075) or total WM SI (β = 0.179, *p* = 0.235).

### Associations between HPτ/Aβ burden and WM SVD with WMH in cases with minimal cortical neurodegenerative pathology

**Fig. 4:**
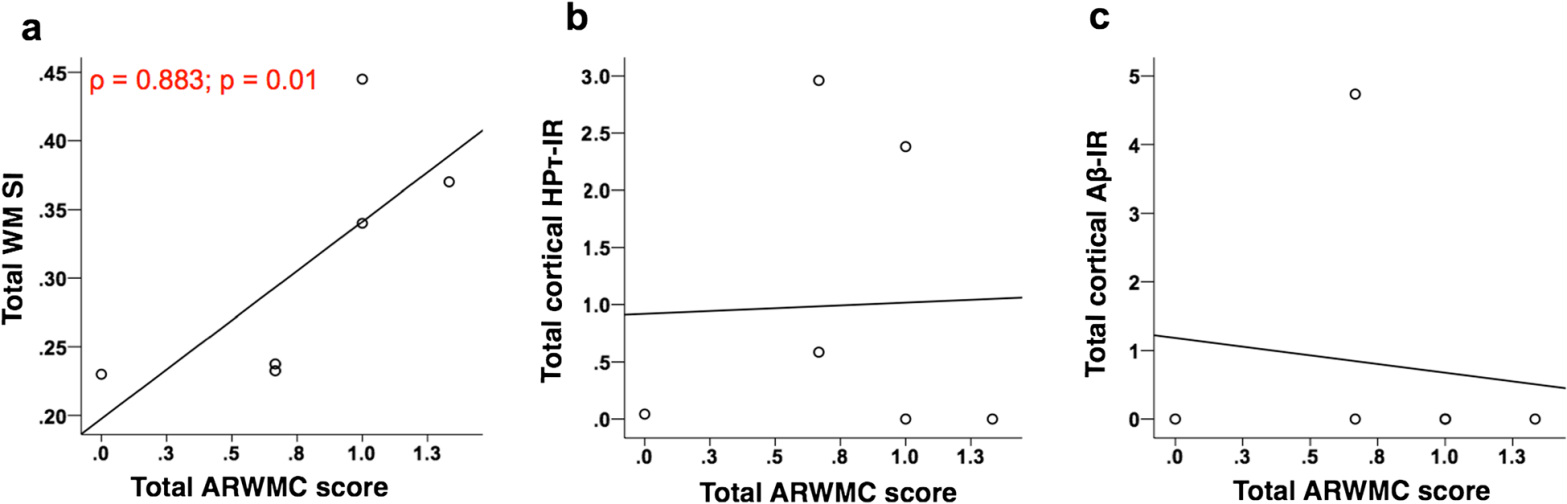
Correlations between total white matter sclerotic index (**a**) (WM SI; measure of small vessel disease severity) as well as total cortical percentage area covered by HPτ-IR (**b**) (AT8 antibody for hyperphosphorylated tau burden) and 4G8 (**c**) immunoreactivity (Aβ-IR; for amyloid-β depositions/plaques) with age related white matter change score (ARWMC; measure for white matter hyperintensities) in cases with minimal hyperphosphorylated tau pathology (classified as Braak NFT stage 0-II [6]). Only total WM SI (**a**) correlated with total ARWMC score; no correlation was seen between total cortical HPτ-IR and Aβ-IR with total ARWMC scores. Only significant p values (and associated correlation coefficients (ρ)) are shown

To investigate whether the association between WM SVD and WMH in cases with minimal cortical HP-τ burden differs from the one observed in the entire study cohort (of which 63.9 % were AD cases with moderate to high cortical HP-τ burden), we restricted the analysis to control cases classified as Braak NFT stage 0-II (*n* = 6), which exhibit no or only minimal cortical HP-τ pathology. We investigated associations between total cortical HPτ/Aβ burden, total WM SVD severity and age at death with total WMH scores. No correlation was seen between age at death and total ARMWC score (ρ = 0.235; *p* = 0.327), therefore, Spearman’s and Pearson’s correlation coefficients (one tailed) were employed. Here, the correlation between total WM SI and total ARWMC score appears much stronger (ρ = 0.883; *p* = 0.01; Fig. 4a) than the one observed for the entire study cohort (r' = 0.311, *p* = 0.035; Fig. 3o), which is driven only by the correlation in the occipital region. Additionally, no significant correlations were seen between both total cortical HPτ-IR (ρ = −0.433; *p* = 0.196 Fig. 4b) and total cortical Aβ-IR (ρ = 0.27; *p* = 0.303; Fig. 4c) with total ARWMC score.

## Discussion

Here, we demonstrate that in frontal, temporal and parietal regions, as well as in the entire hemisphere, cortical HPτ burden predicted the severity of WMH independent of both cortical Aβ burden and WM SVD severity. However, in cases virtually lacking cortical HPτ pathology we found a strong correlation between the severities of SVD and WMH.

WMH is a descriptive term for diffuse, low-density changes of the cerebral WM as seen on T2-weighted MRI images. Despite the assumption that WMH are the result of SVD, the underlying pathogenesis is unclear and appears to be associated with a heterogeneous mixture of vascular and degenerative processes as determined by previous studies [24, 32, 33, 62], and our data that demonstrated increasing WMH severity with increasing cortical HPτ and Aβ pathology, SVD severity, and age. As expected, age at death was associated with increasing WMH severity as age is one of the strongest risk factors related to the development of WMH [8], as well as being associated with increasing AD-related pathology [11] and dementia. In agreement with previous studies [4, 47, 50] we found significantly more severe WMH in AD i.e., cognitively impaired cases compared to controls, however, no respective differences were seen with regard to the severity of SVD suggesting that SVD may not be the main underlying cause for WMH in AD. Moreover, only cortical HPτ pathology independently predicted the severity of WMH when calculations were performed for frontal, temporal and parietal regions, as well as the entire hemisphere. These findings further point towards an important role of increasing amounts of cortical HPτ pathology in the pathogenesis of WMH. It is important to note that the accumulation of AD-pathology frequently occurs in normal aged individuals without compromising cognitive function [5, 23, 25, 56], therefore, the impact of HPτ pathology on WM damage may not be restricted to just patients that exhibit cognitive decline or dementia.

This association between increasing HPτ pathology and increasing WMH severity corroborates previous neuropathological-imaging studies that reported an association between increasing Braak NFT stage and increasing WMH severity [13, 24]. Furthermore, tau protein has been implicated in the clinical conversion from MCI to AD as shown in a recent study by Tosto and colleagues, who demonstrated that in MCI participants with high cerebral spinal fluid level of total-tau, higher parietal WMH volume predicted conversion to AD [59]. In addition, our data implicating HPτ pathology as a predictor for WMH severity in the temporal and parietal WM confirms neuropathological studies reporting WM damage in AD to predominately affect the temporal [12] and parietal [35] WM. However, these studies were based on semi-quantitative assessment that provides only a crude estimation of pathological burden [6, 55], while our study implemented quantitative assessment that better reflects the actual amount of pathology and seems better suited to identify associations between different types of pathological lesions.

Despite our data indicating WM damage in cases with cognitive impairment is associated with HPτ pathology, the underlying mechanism of how HPτ causes axonal loss remains unclear. Two possible pathomechanisms have been proposed; firstly, neuronal death is associated with the accumulation and deposition of HPτ pathology [19], which may lead to the disintegration of associated axons. Secondly, in neurodegenerative diseases with protein aggregation and deposition, including AD, major synaptic and axonal loss can precede the loss of the neuronal soma [10, 49]. This type of axonal loss, which is independent of neuronal death, is thought to be the result of dysfunction and/or blockage of fast anterograde axonal transport [9, 42]. With respect to the development of HPτ pathology, the hyperphosphorylation of the microtubule-associated tau protein leads to the destabilization of the principal cytoskeletal component microtubules that are critical for fast anterograde transport in axons [36]. Impairment of axonal transport is thought to activate the cysteine protease calpain, resulting in the retrograde degradation of axonal cytoskeletal proteins and subsequent loss of the axon fibre [9, 36]. Both of these suggested mechanisms might explain our finding of increasing cortical HPτ burden independently predicting the severity of WMH.

In cases with minimal HPτ pathology, categorized as Braak NFT stage 0-II, we found a strong correlation between increasing WM SVD and WMH severities confirming previous pathologic and imaging studies demonstrating an association between SVD and WM damage [45, 52]. Of note, the small sample size of six cases was a limitation and repeat studies are warranted using a larger cohort. This finding is also in agreement with a number of recent contributions that point to the independence of WMH from classical AD biomarkers [27, 29, 43]. On the other hand, in the entire study cohort, cases with higher HPτ pathology burden and cognitive impairment exhibited significantly higher ARWMC scores compared to controls, while no significant differences were seen in the severity of WM SVD. Moreover, the severity of WM SVD failed to predict WMH score. Taken together, our findings suggest that in general, both cortical HPτ pathology and WM SVD may lead to the development of WMH; however, in neurodegenerative diseases such as AD, WMH are primarily associated with cortical HPτ pathology, while WM SVD may be an additional contributing factor. On the other hand, in cases virtually lacking cortical HPτ pathology, WM SVD seems to play an important role in the development of WMH. SVD is complex and heterogeneous and our assessment was limited to the measurement of the vessel wall thickness i.e., measure of fibrosis only, therefore, future studies may warrant assessment of other SVD-related pathologies, e.g., blood brain barrier breakdown and perivascular space enlargement. Additionally, we did not assess other possible causes of ischemic damage such as cerebral amyloid antipathy, orthostatic hypotension, and myocardial infarction.

Our study incorporated the use of *post-mortem* T2 weighted MR imaging for the assessment of WMH. There is currently limited data directly comparing human *in vivo* and *post-mortem* MRI imaging of WMH and the effects of fixation on MR characteristics. One such study by Macchi and colleagues compared MS lesions *in vivo* and *post-mortem* MRI scan from a single MS patient and determined that the MRI signal and contrast from the *post-mortem* scan was satisfactory compared to the *in vivo* scan [37]. Furthermore, an investigation into the effects of fixation on quantitative MRI of human brain slices revealed there were no significant changes detected in WMH and normal appearing WM after fixation [53]. Further studies are warranted to elucidate any significant alteration of MR characteristics as a result of the fixation process. Nevertheless, MRI and histopathology comparative studies have previously shown that *post-mortem* MRI of formalin fixed tissue is a reliable method to obtain data on both the severity and distribution of WM damage [14, 15, 38, 52].

Neuroimaging is emerging as an important biomarker in the diagnosis of pre-symptomatic AD and multiple studies have indicated regional specific WM damage in AD patients compared to normal ageing. Reduced fractional anisotropy (indicative of reduced tissue integrity) on diffusion tensor imaging (DTI) is frequently seen in the WM of the posterior regions, i.e., parietal, temporal and occipital lobes [1, 3, 4], as well as the major fiber bundles, i.e., inferior and superior longitudinal fasciculi [34], the parahippocampus [44] and corpus callosum [4]. Previous neuroimaging studies have demonstrated associations between increasing changes in WM and cortical atrophy in AD [1, 3], which could possibly be explained by WM damage resulting from axonal loss caused by HPτ-related neuronal death.

## Conclusions

Clinical diagnosis and decision making in dementia is partly based on neuroimaging e.g., medial temporal lobe atrophy is associated with AD [28], while WMH are usually regarded as an indicator of vascular cognitive impairment/dementia [48]. However, given that AD is the most common cause of age-associated dementia [57], our findings strongly suggest that WMH in cognitively impaired individuals rather indicate cortical AD associated neurodegenerative pathology than SVD/CVD. Hence, current interpretation of WM neuroimaging may result in inadequate management of patients and be detrimental for the stratification of patient cohorts in clinical trials. Further studies are warranted to better determine the underlying pathological processes that may lead to WMH.
